# Design and optimization of CIGS-based solar cell with surface dielectric nanostructures arrangement

**DOI:** 10.1038/s41598-025-34836-0

**Published:** 2026-01-27

**Authors:** Fatma M. Abdel Hamied, Roaa I. Mubarak, K. R. Mahmoud, Mohamed Farhat O. Hameed, S. S. A. Obayya, R. El-Bashar

**Affiliations:** 1 Faculty of Engineering, Electronics and Communications Department, Helwan University, Cairo, Egypt; 2 National Telecommunications Regulatory Authority, Ministry of Communication and Information Technology, Giza, Egypt; 3 Center for Nanotechnology, Zewail City of Science, Technology and Innovation, October Gardens, 6th of October City, Giza, 12578 Egypt; 4 Academy of Scientific Research and Technology (ASRT), Cairo, Egypt; 5 Faculty of Engineering, Mathematics and Engineering Physics Department, University of Mansoura, Mansoura, 35516 Egypt; 6 Centre for Photonics and Smart Materials, Zewail City of Science, Technology and Innovation, October Gardens, 6th of October City, Giza, 12578 Egypt; 7 Faculty of Engineering, Electronics and Communications Department, University of Mansoura, Mansoura, 35516 Egypt; 8 National Institute of Laser Enhanced Sciences (NILES), Cairo University, Giza, 12613 Egypt

**Keywords:** Thin-film solar cells (TFSCs), Dielectric nano-particles, Copper indium gallium selenide (CIGS), Finite-difference time-domain (FDTD), Anti-reflection coating (ARC), Light absorption, Engineering, Materials science, Nanoscience and technology, Optics and photonics, Physics

## Abstract

This study introduces a novel design of copper indium gallium selenide (CIGS) thin-film solar cells by incorporating aluminum arsenide (AlAs) dielectric nano-particles on the front surface. Three nanoparticle geometries-cubic, cylindrical, and spherical—are explored to enable broadband light absorption and enhance overall device efficiency. The optimization of structural parameters is performed using the particle swarm optimization (PSO) algorithm in conjunction with the Lumerical finite-difference time-domain (FDTD) solver. Simulation results demonstrate that the cubic nanoparticle design delivers the highest performance, achieving an average absorption of 93.5%, corresponding to 31.7% improvement over the baseline cell. The enhanced performance of cubic AlAs nano-particles arises from their support of broadband, high-order Mie resonances, enabled by sharp edges and flat facets. Considering recombination mechanisms, the power conversion efficiencies (PCEs) of the proposed cubic-based structures are enhanced to 17.6%, compared to the conventional design of 12.56%. The reported surface-integrated dielectric nanostructure approach demonstrates strong potential for high-efficiency thin-film solar cells (TFSCs) with reduced material usage, lower fabrication complexity, and cost-effectiveness.

## Introduction

The rising global energy demand, fueled by industrial growth and population increase, has heightened the urgency for dependable and sustainable energy solutions^1^. Among renewable options, solar energy stands out for its wide availability, sustainability, and economic viability^2^. Photovoltaic (PV) solar cell (SC) technology is widely used but still requires advancement to produce low-cost, lightweight, and high-efficiency device^3,4^. Currently, the majority of commercial SCs are based on crystalline silicon (c-Si), which, despite its abundance and suitability, remains costly due to complex crystallization processes^5,6^. To address this problem, TFSCs have been developed, featuring a significantly reduced absorber layer thickness^7,8^, yielding lighter, more flexible, and cost-effective devices^9^. However, their major limitation lies in the reduced light absorption, particularly at longer wavelengths, resulting in lower PCE compared to bulk crystalline counterparts^10,11^. To this end, various light-trapping strategies have been developed, including the integration of metallic or dielectric nano-particles^12^, nano-antennas (NAs)^13^, nano-wires^14,15^, grating nanostructures^16–18^, and plasmonic back reflectors. Specifically, metallic nano-particles^19^ can support localized surface plasmon (LSP) resonances, leading to enhanced light absorption, but suffer from Ohmic losses and stability issues. In response, recent research has shifted toward dielectric resonant nanostructures, which offer a promising alternative with reduced losses and improved long-term stability^20,21^. In this regard, Ferhati et al.^22^ conducted a numerically study on a ZnO/Si heterojunction solar cell incorporating Ag NPs and interface texturization to improve optical absorption. The optimized structure achieved up to 50% enhancement in total absorbance efficiency compared to the planar structure, demonstrating the effectiveness of metallic nanoparticles and surface patterning in boosting light confinement and overall device performance. In a subsequent work, a Se-based TFSC was reported^23^ incorporating Ti and Au metallic sublayers together with Au NPs, achieving 14.7% efficiency as a result of enhanced plasmonic light trapping and better carrier collection. In addition, Elrabiaey et al.^24^ proposed a thin c-Si TFSC with embedded dielectric nanowires, achieving a J_ph_ of 32.8 mA/cm²— 82.2% increase over the conventional 18 mA/cm². Further, Mohsin et al.^2^ utilized a plasmonic nano-particles array combined with an ARC and aluminum reflective layers to improve the light trapping. This approach led to an overall efficiency of 13.3%, reflecting 80.4% improvement compared to conventional TFSCs. Furthermore, the light trapping technique has been achieved by embedding an array of core/shell nano-rod in perovskite SC. Such structure resulted in achieving conversion efficiency$\:({\upeta\:}$) of 19.3%. In 2022^25^, semiconductor nanoparticle arrays on thin-film GaAs solar cells, improving efficiency by 10% over aluminum NPs and by 21% and 30% compared to cells with and without ARC, respectively. Maoucha et al.^26^ presented a lead-free perovskite/SnS tandem solar cell enhanced with plasmonic Au NPs for improved light trapping. Numerical analysis revealed that incorporating SnO₂ and CuO as charge transport layers optimized band alignment, reduced recombination, and achieved 20.1% efficiency. As well, Shaghouli et al.^21^ reported a hybrid design combining a silver fractal pattern atop the active layer with leaky-wave optical NAs beneath the absorber layer, achieved J_SC_ enhancement factors of nearly 1.9. Further, an InP TFSC has been designed with embedded plasmonic Ti NPs, achieving ~ 99% absorption, J_SC_ = 32 mA/cm², V_OC_ = 1.05 V, and η = 29.6%^27^. However, the complex fabrication and unaccounted parasitic plasmonic losses may have led to overestimated performance. Furthermore, T. Ahmed and M. K. Das^28^ enhanced TFSC performance by integrating front-side nanotexturing with rear-side amorphous Si nanowires, reducing reflection and improving light scattering. The design achieved 13.62% efficiency, 34.7% improvement over flat TFSCs.

CIGS solar cells have emerged as a strong alternative to silicon-based PVs, achieving comparable efficiencies with thinner absorber layers owing to their tunable bandgap (1.04–1.7 eV) and high absorption coefficient (~ 10⁵ cm⁻¹)^29–31^. The CIGS solar cells are considered cost-effective because it requires significantly less raw material and enables thin-film deposition on flexible, inexpensive substrates^32,33^. Although certain fabrication techniques, such as vacuum deposition or selenization, may appear costly at the laboratory scale, CIGS technology remains inherently economical due to its thin absorber layer (~ 1–2 μm), low material consumption, and compatibility with low-temperature, large-area, and roll-to-roll processing^34,35^. Industrial demonstrations by companies such as Solar Frontier and Avancis have achieved competitive module costs of approximately $0.25–$0.35/W, comparable to crystalline silicon technologies^35^. However, the limited absorber thickness still leads to insufficient photon absorption at longer wavelengths^31,36^. This work presents an optimized CIGS TFSC design enhanced with AlAs dielectric nanoparticles of cubic, cylindrical, and spherical shapes on the front surface. Using 3D FDTD and PSO optimization, the study evaluates and optimizes light absorption, photocurrent, and efficiency. The cubic nanoparticle design achieves the best optical and electrical performance, with near-perfect absorption (> 97%) and 31.7% enhancement over the baseline. It also yields the highest J_SC_ (37.84 mA/cm²) and efficiency (17.6%), representing 37.8% and 40.3% improvements, respectively. The design remains polarization-insensitive and fabrication-friendly, as the nanostructures are confined to the cell surface.

## Design considerations and numerical methods

### Design considerations

**Fig. 1:**
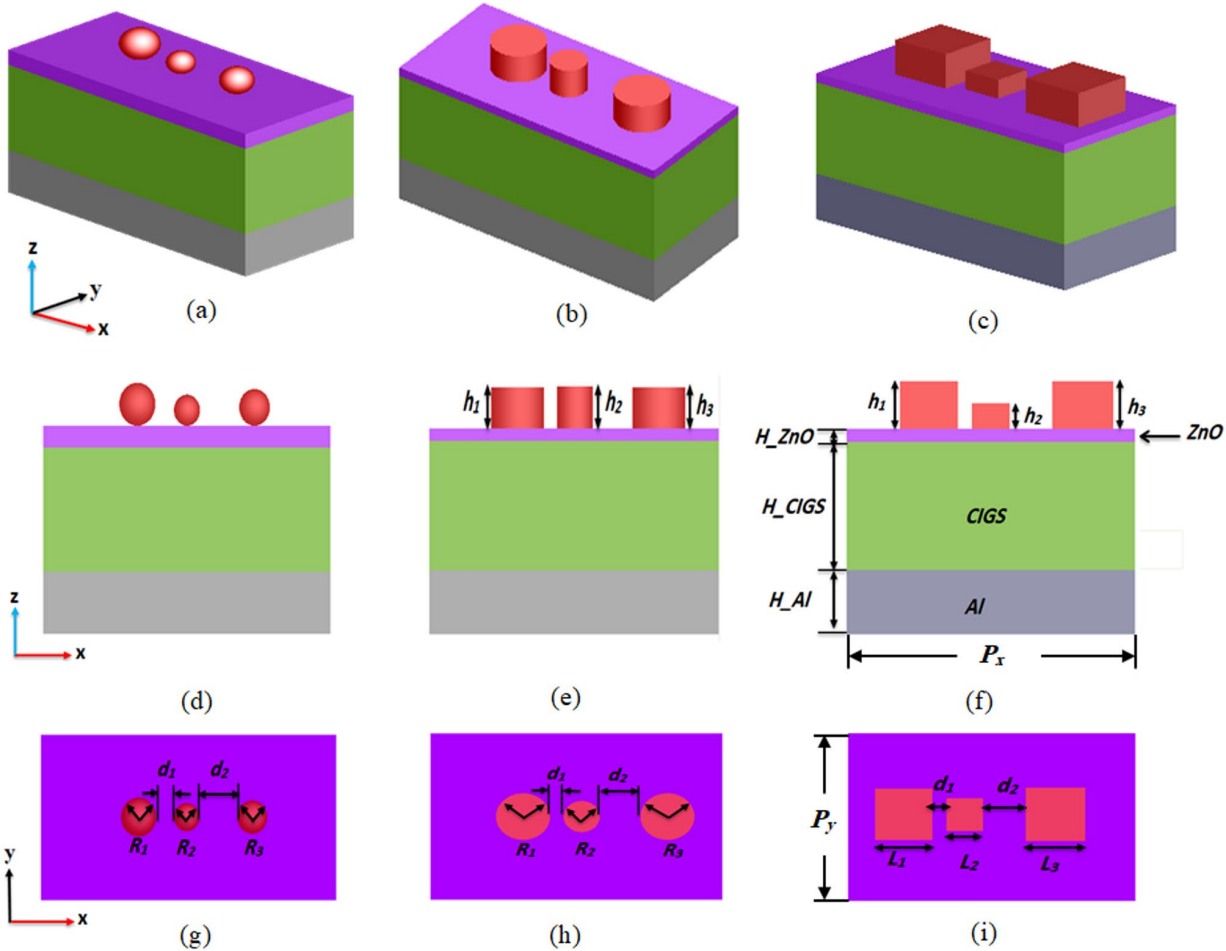
(**a**) 3D view of the proposed CIGS TFSC with the (**a**) spherical (**b**) cylindrical and (**c**) cubic dielectric nanoparticles. The side/top views of the (**d**)/(**g**) spherical-shaped, (**e**)/(**h**) cylindrical-shaped, and (**f**)/(**i**) cubical-shaped nanostructures on top of the solar cell are also shown in Fig. 1.

In this work, top dielectric nano-particles are introduced to enhance light trapping within the absorber, thereby improving photon absorption and boosting conversion efficiency. In the proposed design as shown in Fig. 1, schematic diagrams of the proposed CIGS-based SC integrated with spherical, cylindrical and cubic top dielectric nanostructures. The corresponding side views | top views are shown in Fig. 1(d) | (g), (e) | (h), and (f) | (i), respectively. The conventional SC architecture comprises three primary layers: the active layer, a metallic back contact, and an ARC^20^. In this regard, the proposed CIGS-based SC structure consists of a bottom-to-top sequence of Al/CIGS/ZnO with AlAs dielectric nano-particles positioned on the top. The top ZnO electrode also functions as an ARC, which significantly reduces the reflection and enhance the overall light absorption^37,38^. The Top AlAs dielectric nano-particles help improve the light confinement and extend the absorption bandwidth. The proposed nano-particles configuration utilized are three elements with different inter-element spacing and different dimensions.

In this respect, PSO technique is implemented with FDTD method for maximum integrated light absorption. As shown in Fig. 1(d) and (g), the spherical NPs are defined by a smaller set of parameters, where only radii (R_1_, R_2_, R_3_), and spacing (d_1_, d_2_) are optimized. In contrast, the cylindrical NPs configuration, depicted in Fig. 1(e) and (h), requires the adjustment of radii (R_1_, R_2_, R_3_), heights (h_1_, h_2_, h_3_), and horizontal distances (d_1_, d_2_) to achieve optimal performance. Lastly, the cubic nanoparticle array, illustrated in Fig. 1(f) and (i), involves the optimization of more geometric parameters, including the edge lengths (L_1_, L_2_, L_3_), heights (h_1_, h_2_, h_3_), and inter-element spacing (d_1_, d_2_). CIGS and Al thicknesses are fixed at 400 nm and 200 nm, respectively, throughout the optimization. The period (*P*_*x*_ *× P*_*y*_*)* of the suggested nanoparticles is set to 1000 nm × 500 nm. The solar cell features a moderately n-doped top layer and a heavily p-doped CIGS back layer, forming an internal electric field that facilitates efficient charge separation. As photons are absorbed in the CIGS layer, electron–hole pairs are generated and driven by this field electrons migrate toward the front contact and holes toward the back resulting in current generation^39^. These proposed designs are optically simulated using computational domain of 1000 nm × 500 nm with height of 2 μm with minimum mesh size of 5 nm.

### 2.2 numerical methods and discussion

To investigate the performance of the proposed SC structure, a combination of optical and electrical simulation studies are employed. These simulations are used to extract critical performance metrics. The optical simulation focuses on computing optical characteristics of absorption, reflection, transmission, and photocurrent density (Jₚₕ). Using the FDTD method, the optical characteristics are accomplished via the Lumerical software suite. This approach numerically solves Maxwell’s equations to simulate the interaction of electromagnetic waves with the device architecture. In this regard, the material optical parameters of aluminum (Al) are sourced from the Ansys/Lumerical material database, relying on Palik’s empirical data^40^. For the proposed CIGS, Zinc oxide (ZnO) and aluminum arsenide (AlAs), the optical constants are incorporated from well-established sources in the literature^41–43^. The bandgap of the CIGS-based active layer is equal to 1.07 eV^31^. In this respect, a plane wave source of wavelengths from 300 nm to 1100 nm, corresponding to the CIGS bandgap, is utilized to simulate the solar spectrum. To simulate the proposed periodic array structure, the computational domain is defined with periodic boundary conditions along the x- and y-axes. Perfectly matched layers (PMLs) are applied along the z-direction to absorb outgoing radiation and prevent artificial reflections^44^. In this study, a minimum mesh size of 5 nm is applied in z-direction to ensure accurate spatial resolution throughout the simulation domain. The Al back reflector is taken in this work by 200 nm-thick. Therefore, it acts as a perfect reflector, ( i.e. T(λ) ~ 0), and the absorption A(λ) can be calculated as follows^45^:

1 $$A(\lambda )\,=\,1\, - \,R(\lambda )$$

where R(λ) is the reflection as a function of wavelength λ. The PSO technique is incorporated in this research to optimize the light absorption (A($\:{\uplambda\:}$)) and thus the design efficiency. In this investigation, the integrated absorption serves as the fitness criterion, guiding the search for the optimal geometric configuration. To explore the device electrical characteristics, the optical generation profile is exported to the electrical solver with considering the optimal device geometry. The CHARGE solver is employed to simulate the transport and collection of photo-generated carriers, enabling accurate calculation of J_sc_ and power conversion efficiency (η). The results are obtained by solving the coupled drift-diffusion and Poisson equations, which govern carrier dynamics under steady-state conditions. FF denotes the fill factor, which reflects the squareness of the current-voltage (I–V) curve and is given by^46^:

2 $$\:\boldsymbol{F}\boldsymbol{F}\:=\:\frac{{\boldsymbol{P}}_{\boldsymbol{m}\boldsymbol{a}\boldsymbol{x}}}{{\boldsymbol{J}}_{\boldsymbol{S}\boldsymbol{C}}\:{\boldsymbol{V}}_{\boldsymbol{o}\boldsymbol{c}}}$$

In this expression, $\:{\mathrm{P}}_{\mathrm{m}\mathrm{a}\mathrm{x}}$ is the maximum electrical power delivered by the SC, V_OC_ is the maximum voltage available from a solar cell when no external load is connected, and is calculated using^47^:

3 $$\:{\:\:\:\:\boldsymbol{V}}_{\boldsymbol{o}\boldsymbol{c}}=\:\frac{{\boldsymbol{K}}_{\boldsymbol{B}}{\boldsymbol{T}}_{\boldsymbol{a}}}{\boldsymbol{e}}\:\boldsymbol{l}\boldsymbol{n}\left(\:\frac{{\boldsymbol{J}}_{\boldsymbol{S}\boldsymbol{C}}}{{\boldsymbol{J}}_{0}}+1\right)$$

where $\:{K}_{B\:}\mathrm{i}\mathrm{s}\:\mathrm{B}\mathrm{o}\mathrm{l}\mathrm{t}\mathrm{z}\mathrm{m}\mathrm{a}\mathrm{n}\:\mathrm{c}\mathrm{o}\mathrm{n}\mathrm{s}\mathrm{t}\mathrm{a}\mathrm{n}\mathrm{t},$ $\:\:\:{\mathrm{T}}_{\mathrm{a}}$ is the absolute temperature, and $\:{\mathrm{J}}_{0}$ is the dark saturation current.

**Fig. 2:**
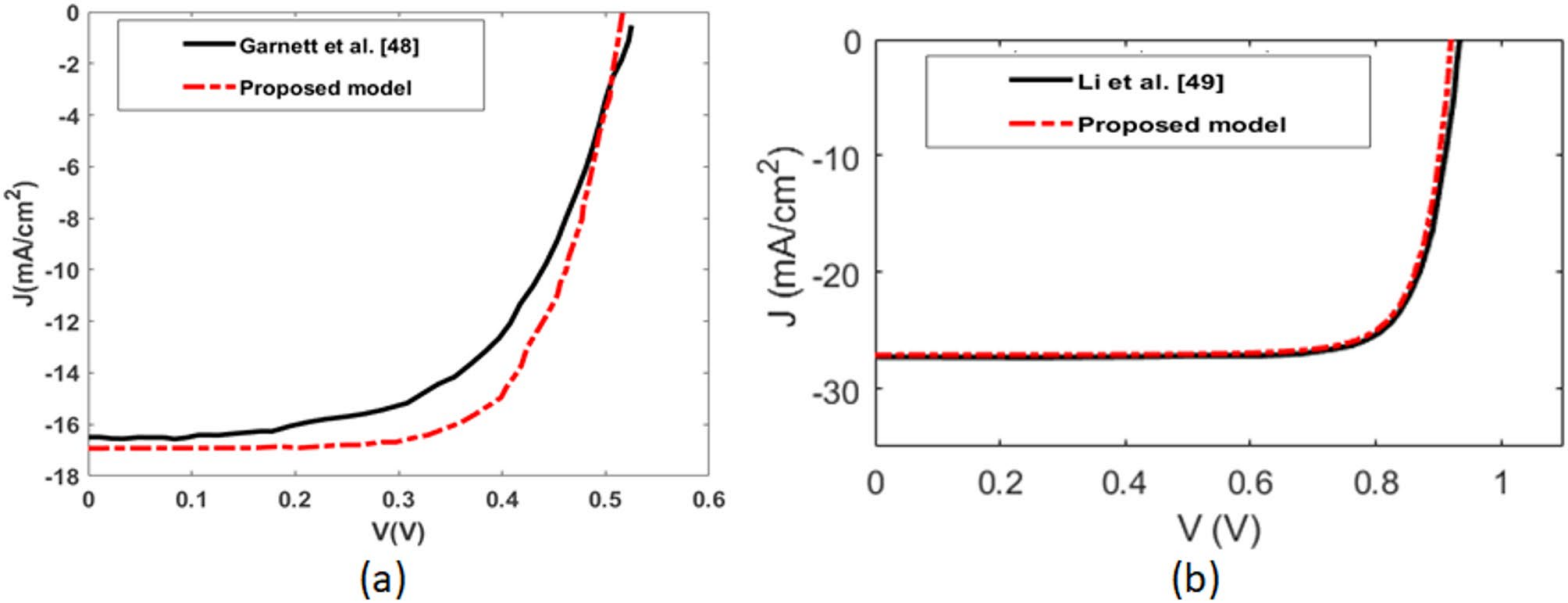
Comparison of the I–V characteristics of the proposed model and (**a**) Experimentally measured data for a silicon nanowire solar cell^48^, (**b**) COMSOL numerical results reported in Ref^49^.

To check the validity of the numerical results, initially, the solver was calibrated against previously reported experimental data from a study on silicon hexagonal nanowire (SiNW) solar cell structure^48^. A comparison between the simulated I–V curve and the reported experimental results is presented in the Fig. 2(a), showing a good agreement. The structure consists of silicon pillars with a diameter of 390 nm, a height of 5 μm, and a pitch of 530 nm, arranged in a hexagonal lattice on a silicon substrate. Although the Si wafer thickness in the reference study varied from 8 to 20 μm, most of the incident light was absorbed within the nanowire array itself. For the present simulations, a 1 μm-thick silicon substrate was employed. The model was implemented in 3D, illuminated using a plane wave source, and utilized symmetry boundary conditions to represent both the periodicity and symmetry of the nanowire array. To investigate the average I-V of the proposed design, simulations were conducted for two orthogonal polarizations. The CHARGE solver was then used to model the complete optoelectronic behavior of the device. The optical generation rate profiles from the optical simulation were imported into the drift–diffusion equations within the CHARGE module, with each generation rate scaled by 0.5, as required. The structural and doping parameters were kept consistent with those reported in the reference design. The results confirm a good agreement between the simulated J–V characteristics and the experimental data presented in Ref^48^. , validating the accuracy and reliability of the CHARGE solver for this work.

In addition to the validation performed against a previously reported experimental data, another validation was conducted using a previously published simulated data to ensure the effectiveness of the electrical characterization model. Specifically, the I–V characteristics obtained from the CHARGE module of Lumerical software were compared with those reported in a published numerical study of a GaAs nanowire (NW) solar cell^49^, as shown in the Fig. 2(b). In this reference design^49^, the solar cell consists of GaAs nanowires with an 80 nm diameter and 2 μm height, arranged in a periodic lattice with a pitch of 291 nm, grown on a 196 nm GaAs substrate. The surface recombination velocity (SRV) is taken by 0 m/s with carrier lifetime of 800 ps. In this work, the simulation region is 3D, and a plane wave source is used for illumination. Symmetric boundary conditions are used to account for the periodicity as well as the symmetry of the design. The CHARGE solver can be used to characterize the complete optoelectronic response of the solar cell. To obtain the J-V characteristics under illumination, the optical generation profile file is imported from the optical solver to the drift-diffusion equations of the CHARGE solver. The structure and doping profile follow the reference design of axial and radial shell thickness of 10 nm and 110 nm, respectively. The donor doping of the core and substrate is equal to 5$\:\times\:$10^18^ cm^− 3^ and the acceptor doping of shell is equal 5$\:\times\:$10^18^ cm^− 3^. Figure 2(b) shows the J-Characteristics of the CHRAGE Lumerical solver compared to the published one using the Comsol Multi-physics Software in^49^. It is evident that there is an agreement in between the CHARGE solver simulation result and the published one of Ref^49^.

In this investigation, The PSO-based optimization numerical method is utilized to optimize the nanostructures geometries to maximize light absorption as computed by FDTD. PSO begins by defining the number of swarm particles, which represent possible solutions, and the number of iterations to be executed, as well as the decision space that specifies the allowable range of each design parameter. Once these parameters are set, the particles are initialized at random positions within the search space, forming the initial population of potential solutions. Then, the optical performance is evaluated of each particle using 3D FDTD by computing the absorption spectrum A(λ) over 300–1100 nm, with the fitness function defined as the average absorption A_m_ as follows:

4 $$\:\mathrm{A}\mathrm{m}=\frac{{\int\:}_{{\boldsymbol{\lambda\:}}_{1}}^{{\boldsymbol{\lambda\:}}_{2}}\boldsymbol{A}\left(\boldsymbol{\lambda\:}\right)\:{\boldsymbol{{\rm\:I}}}_{\boldsymbol{A}\boldsymbol{M}\:1.5\:}\left(\boldsymbol{\lambda\:}\right)\:\boldsymbol{d}\boldsymbol{\lambda\:}\:\:\:\:}{{\int\:}_{{\boldsymbol{\lambda\:}}_{1}}^{{\boldsymbol{\lambda\:}}_{2}}{\boldsymbol{{\rm\:I}}}_{\boldsymbol{A}\boldsymbol{M}\:1.5\:}\left(\boldsymbol{\lambda\:}\right)\:\boldsymbol{d}\boldsymbol{\lambda\:}\:\:\:\:\:\:\:\:\:\:\:\:}$$

where λ_1_ and λ_2_ are the starting and ending wavelengths of the studied range, and $\:{{{\rm\:I}}}_{\mathrm{A}\mathrm{M}\:1.5\:}$represents the solar spectra under AM 1.5 conditions, as defined by the NREL standard.

In this study, the PSO optimization runs over 100 iterations with 10 swarm particles. After evaluating fitness function, the local and global best values are updated. The local best corresponds to the maximum fitness value achieved by an individual particle, while the global best represents the maximum fitness value found among all particles. Whenever a new local best exceeds the current global best, the global best is updated accordingly. If the stopping condition which is either the maximum number of iterations or the convergence of optimal parameters has not been met, the velocity and position of each particle are updated according to the PSO equations^22^, allowing them to explore new regions of the search space. The process of optimization continues in an iterative loop until the termination condition is met. Finally, once the stopping condition is satisfied, the optimum parameters (global best) are obtained, representing the best configuration or set of parameters that yield maximum average absorption. To ensure the designs are suitable for practical fabrication, the final dimensions are approximated to realistic integer values. The initial dimensions along with the corresponding optimized parameters are detailed in Table 1.

**Table 1:** List of the initial and optimized geometrical parameters used for the proposed thin-film CIGS solar cell involving different shapes of dielectric nano-particles.

Parameter	Decision space		Optimum values		
	From	To	Cubic	Cylindrical	Spherical
*L*_*1*_ *(nm)*	*50*	*250*	*200*	*---*	*---*
*L*_*2*_ *(nm)*	*50*	*250*	*130*	*---*	*---*
*L*_*3*_ *(nm)*	*50*	*250*	*210*	*---*	*---*
*d*_*1*_ *(nm)*	*50*	*150*	*50*	*50*	*70*
*d*_*2*_ *(nm)*	*50*	*150*	*150*	*150*	*150*
*h*_*1*_ *(nm)*	*20*	*150*	*150*	*127*	*---*
*h*_*2*_ *(nm)*	*20*	*150*	*82*	*130*	*---*
*h*_*3*_ *(nm)*	*20*	*150*	*150*	*127*	*---*
*R*_*1*_ *(nm)*	*50*	*110*	*---*	*100*	*70*
*R*_*2*_ *(nm)*	*50*	*110*	*---*	*67*	*50*
*R*_*3*_ *(nm)*	*50*	*110*	*---*	*100*	*60*
*H_ZnO(nm)*	*40*	*300*	*40*	*40*	*70*

### Optical characterization

**Fig. 3:**
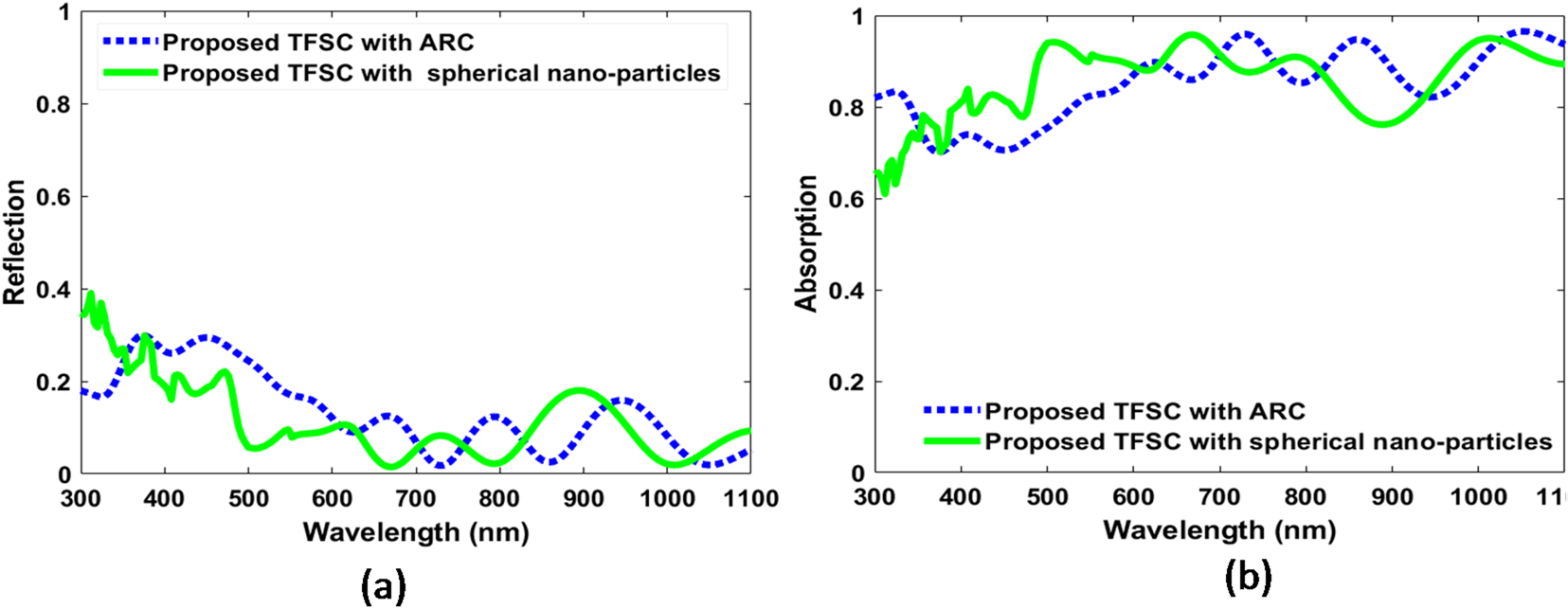
The absorption and reflection within the active layer of the proposed CIGS TFSC with ARC and CIGS TFSC incorporating spherical dielectric nanoparticles atop it before optimization across the wavelength range 300–1100 nm for (**a**) Reflection and (**b**) Absorption.

In this study, we initially explored spherical NPs as a light-trapping enhancement approach to evaluate their effect on short-wavelength light behavior, comparing the SC structure with ARC. As illustrated in Fig. 3, incorporating spherical NPs significantly alter the reflection and absorption spectra, with noticeable improvements in performance. The initial geometrical parameters for spherical NPs are designed with radii of 70 nm, 100 nm, and 70 nm (R_1_, R_2_, and R_3_), and spaced 50 nm apart (d_1_ and d_2_). Therefore, the spherical NPs have a good potential for improving the solar cell efficiency. To further minimize reflection, boost absorption, and reduce material usage, we applied the PSO method, as shown in the following section.

**Fig. 4:**
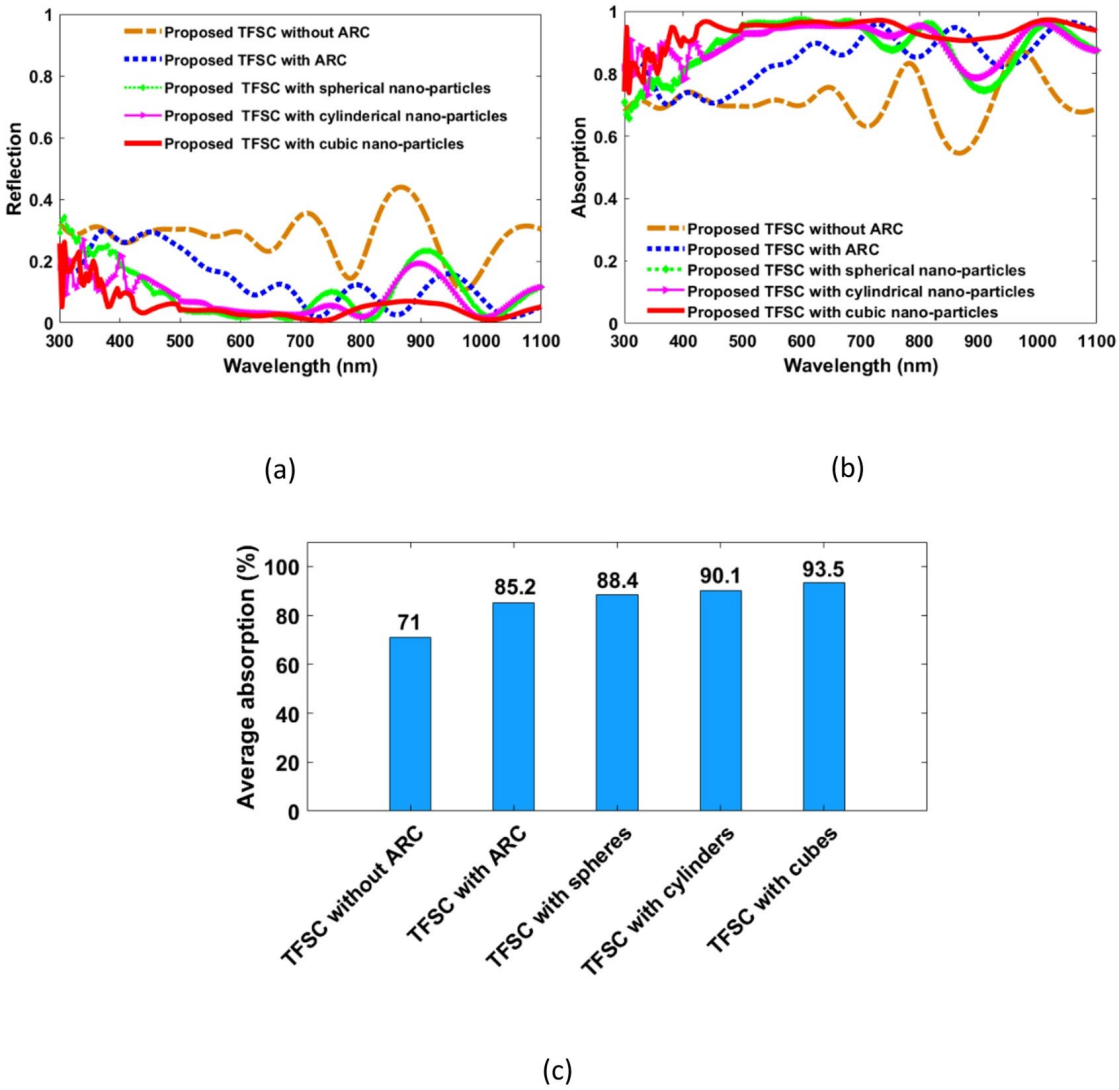
Comparison of the proposed conventional CIGS TFSC without and with ARC, and a CIGS TFSC with cubic, cylindrical and spherical dielectric nanoparticles atop it in the wavelength range 300–1100 nm for both TE and TM polarization (**a**) Reflection, (**b**) Absorption, and (**c**) Average absorption.

After the optimum geometrical parameters have been obtained, the absorption and reflection, J_ph_, and ultimate efficiency of the optimized TFSCs are introduced. Figure 4 shows the wavelength-dependent reflection, absorption, and average absorption of the suggested SC integrated NPs configurations versus the conventional structures with and without the ARC under TE and TM polarizations, respectively. In this study, three different NPs geometries are considered; spherical, cylindrical, and cubic shapes. One can notice that the reflection spectrum of the conventional structure is high due to the high refractive index contrast at the CIGS/air interface. Using the ARC of ZnO material, the reflection spectra are reduced, especially at the longer wavelengths. The mean reflection is reduced to 14.6% with a reduction of 49.65% relative to the conventional structure without ARC. By incorporating the top NPs to the ARC, the reflection is strongly dropped over the broadband spectra as shown in Fig. 4(a). The average reflection is reduced to 11.5%, 9.8% and 6.4% by using the spherical, cylindrical, and cubic shapes, respectively, with a drop of 21.23% | 60.34%, 32.9% | 66.2%, and 56.2% | 77.9%, relative to the conventional design with | without the ARC.

The reported conventional CIGS TFSC, represents a baseline TFSC design, and it contains only the substrate layer Al and the absorber layer CIGS. It can be noted from Fig. 4(b) that the suggested conventional TFSC maintains a consistent light absorption approximately 70% across the wavelength range 300–600 nm. However, light absorption fluctuates between 58% and 87% in the wavelength range of 600–1100 nm, resulting in an average light absorption of 71% over the entire range examined. Upon the addition of the ZnO ARC layer to the conventional CIGS TFSC, there is a notable enhancement in light absorption across the wavelength range of 450–1100 nm, with absorption levels never falls below 70% but increases up to 97%. In this case, the average light absorption in the wavelength ranges of 300 to 1100 nm is increased to 83.4% with an enhancement factor of 17.5% as shown in Fig. 4(c) indicating an improvement in its ability to reduce reflection and capture solar energy. In this work, the integration of top-layer spherical-based AlAs NPs leads to notably improved absorption, especially within the 350–700 nm range, demonstrating an efficient light trapping across the visible spectrum. This enhancement raises the average light absorption to 88.4% with 24.5% increase over the baseline design.

The performance improvement from integrating surface AlAs nanoparticles arises from their low absorption in the visible–NIR range, minimizing parasitic losses while enabling efficient light scattering. Their high refractive index is graded and closely matches that of the underlying ZnO layer. Therefore, they are supporting strong Mie resonances that enhance forward scattering into the CIGS absorber. Further, the light that is trapped inside the dielectric will efficiently couple to the underlying layer. Consequently, these permit increasing light confinement and path length. Additionally, they induce near-field enhancement at the absorber interface, strengthening the local electric field and boosting absorption where CIGS is typically weaker, leading to improved overall efficiency.

The absorption spectra of the proposed design with other NPs geometries are also investigated. One can notice the absorption is further improved for the other studied cylindrical- and cubic-shaped nanosructures relative to the spherical-shaped configuration. We can figure out that the average absorption reaches up 90.1% with 26.9% improvement when cylindrical NPs. Notably, the incorporation of cubic-shaped dielectric NPs achieved near-perfect absorption (> 97%), corresponding to 31.7% increase in average absorption relative to the baseline design. The enhancement with including NPs can be attributed to the geometrical complexity. As the geometrical complexity increases—from spherical to cylindrical to cubic—the nanostructures support richer and higher-order optical resonances, leading to enhanced light trapping and improved device performance. Therefore, the cubic-based geometry offers a reduced light reflection, contributing significantly to the overall enhancement of TFSC efficiency. Despite their larger volume, cubic-shaped dielectric NPs demonstrated the highest overall performance among the studied TFSC configurations. Specifically, the volume of cubic NPs is approximately 1.3 times greater than that of the cylindrical-shaped NPs, and 4.5 times greater than that of the spherical-shaped ones. Interestingly, although spherical NPs occupy less individual volume, their arrangement requires significantly more ARC material, about 1.75 times the ARC volume used for cubic NPs. This indicates that the superior performance of the cubic configuration is not merely a result of increased volume, but rather due to its optimized shape, which enhances light–matter interaction through stronger field confinement and better scattering behavior. Therefore, the cubic geometry offers a more efficient trade-off between material usage and optical performance, making it a favorable design choice for high-efficiency TFSCs.

To illustrate the improved performance associated the proposed structure with cubic NPs, the electric field distributions in the x-z plane along the middle of NPs are investigated. In this study, the electric field profile at the wavelength of 450 nm is examined as it corresponds to the region of maximum optical absorption and closely aligns with the peak of solar irradiance. The absorption spectra of all studied nanostructures demonstrate improved performance compared to the conventional planar design. Notably, the proposed cubic nanoparticles exhibit a pronounced absorption enhancement at shorter wavelengths, with a distinct peak around 450 nm. Therefore, this wavelength was chosen to illustrate and attribute the observed power absorption improvement of the proposed cubic configuration relative to the other studied structures.

**Fig. 5:**
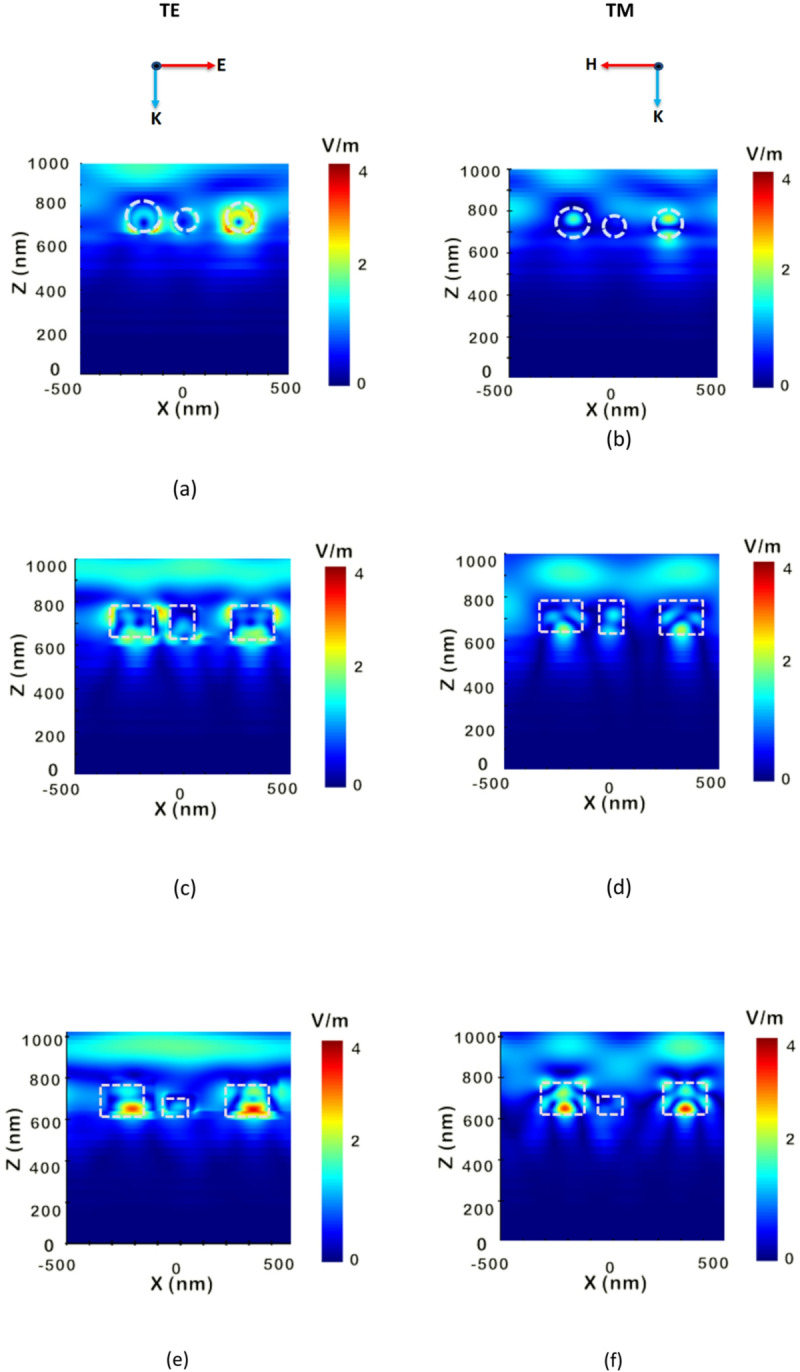
The electric field distributions in x-z plane for the suggested TFSC incorporating spherical, cylindrical, and cubic nano-particles under TE and TM polarizations at a wavelength of 450 nm. (**a**, **b**) Cubic nano-particles under TE and TM polarizations, respectively, (**c**, **d**) Cylindrical nano-particles under TE and TM polarizations, and (**e**, **f**) Spherical nano-particles under TE and TM polarizations.

On the left, Fig. 5(a), (c), and (e) illustrate the electric field distribution for the suggested TFSC integrated with spherical, cylindrical, and cubic NPs under TE polarization, respectively. Meanwhile, on the right, Fig. 5(b), (d), and (f) show the corresponding field profiles for the same NP geometries under TM polarization.

It may be seen that the spherical-shaped NPs design exhibits strong localized confinement near particle edges in TE mode, yet with shallow penetration into the absorber due to their smooth curvature and lack of sharp edges. Under TM mode, the fields are weaker and asymmetrically distributed, limiting light coupling to the active layer and resulting in lower broadband absorption and poorer polarization independence. The cylindrical-shaped NPs provides a broader field spread, with TE mode showing multiple localized hot spots and moderate penetration into the absorber, while TM mode demonstrates better confinement than spherical-shaped NPs but still lacks uniform depth, leading to polarization-sensitive performance. Using the cubic-shaped NPs, the results reveal that the device exhibits the most advantageous characteristics: TE mode generates intense, well-localized hot spots that extend deeply into the absorber, and TM mode achieves nearly identical penetration depth and strength, indicating balanced light coupling across polarizations. This deeper, uniform distribution in cubic-shaped NPs enhances light trapping, increases the optical path length, and maximizes photon absorption, thereby improving carrier generation. This is attributed to the sharp edges and flat surfaces of the cube, which support higher-order Mie modes and enable efficient coupling of light into the active region. The cubic design’s superior performance stems from its enhanced broadband absorption and photocurrent. Therefore, stronger light–matter interaction is achieved with deeper photon penetration for efficient carrier generation throughout the absorber. These findings confirm that cubic nanoparticles deliver the most effective light-trapping among the studied geometries, combining strong near-field enhancement with polarization-insensitive optical behavior.

**Fig. 6:**
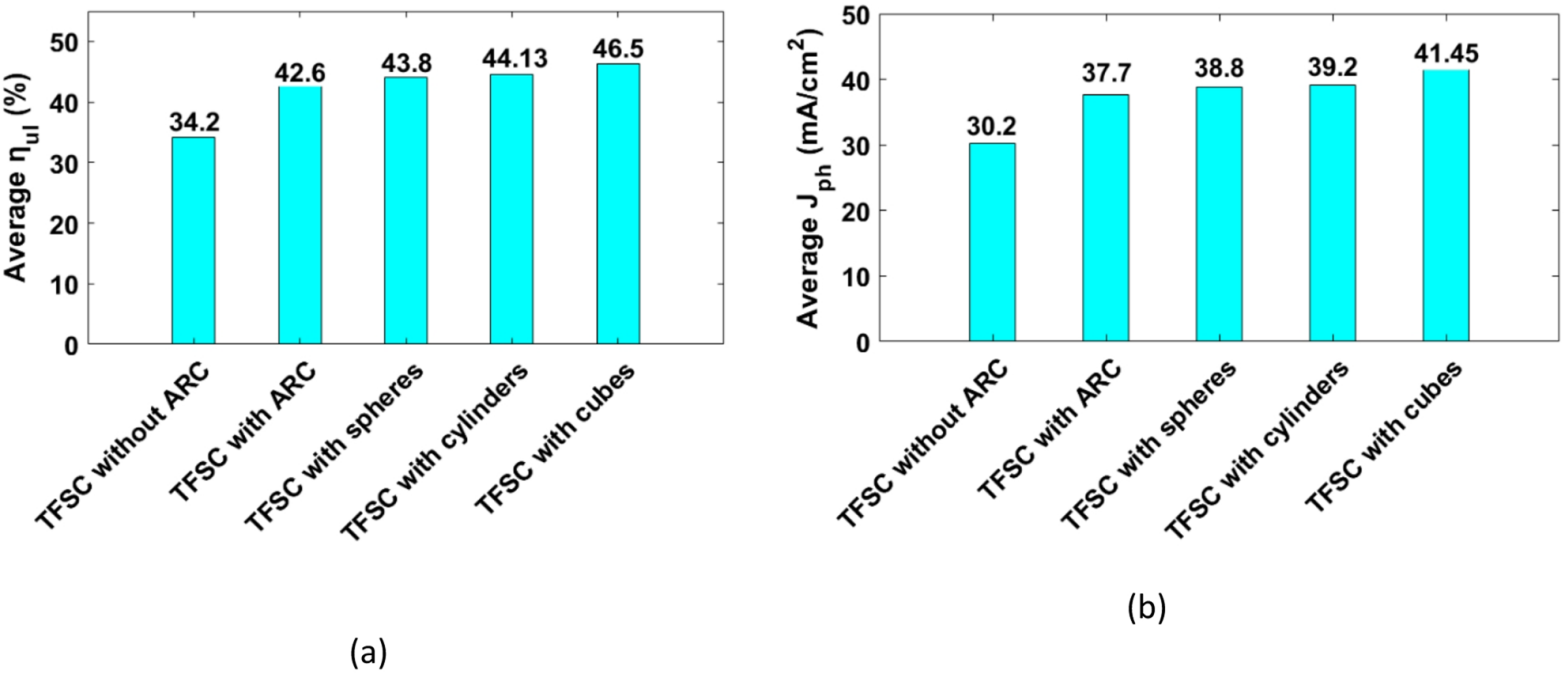
The average ultimate efficiency η_ul_ and photo current density $\:{\mathrm{J}}_{\mathrm{p}\mathrm{h}}$ of the proposed designs, (**a**) η_ul_ and (**b**) $\:{\mathrm{J}}_{\mathrm{p}\mathrm{h}}$.

It is important to recognize that not all absorbed photons contribute effectively to electricity generation. In this context, only the portions of light with photon energies matching the absorber’s bandgap are effectively utilized, while any excess energy is dissipated as heat^3^. The ultimate optical efficiency (η_ul_) quantifies the portion of absorbed optical power that can theoretically be converted into electrical energy at the bandgap limit^50^. Figure 6(a) and (b) shows the η_ul_ and the corresponding J_ph_ for the studied TFSC designs. For the proposed conventional TFSC without ARC, η_ul_ and the corresponding J_ph_ are equal to 34.2% and 30.2 mA/cm^2^, respectively. These values increase dramatically when including the ARC to 42.6% and 37.7 mA/cm^2^ for both the TE and TM polarizations. By incorporating the suggested dielectric NPs, further improvement is accomplished. Therefore, the J_ph_ of the spherical-shaped NPs is improved to 38.8 mA/cm^2^ with corresponding η_ul_ values of 43.8% for both polarizations, respectively. In this regard, the cylindrical-shaped NPs, raises the J_ph_ to 39.2 mA/cm² and the cubical-shaped to 41.5 mA/cm² under both polarizations. The corresponding η_ul_ of the aforementioned geometries elevated to 44% (TE) /44.26% (TM) and 46.5% (TE) / 46.46% (TM), respectively. The averaged ultimate efficiency (($\:{{\upeta\:}}_{\mathrm{u}\mathrm{l}}^{\mathrm{T}\mathrm{E}}+{{\upeta\:}}_{\mathrm{u}\mathrm{l}}^{\mathrm{T}\mathrm{M}})/2$) and photocurrent density (($\:{\mathrm{J}}_{\mathrm{p}\mathrm{h}}^{\mathrm{T}\mathrm{E}}+{\mathrm{J}}_{\mathrm{p}\mathrm{h}}^{\mathrm{T}\mathrm{M}})/2$)) for the conventional TFSC, the TFSC with ARC, and the designs containing cubic, cylindrical, and spherical nanoparticles throughout the 300–1100 nm wavelength range are presented in Fig. 6. The results clearly show that the device with cubic NPs design delivers the most significant improvement. It increases the average η_ul_ by 35.96% and J_ph_ by 37.25% compared to the conventional TFSC lacking an ARC.

**Fig. 7:**
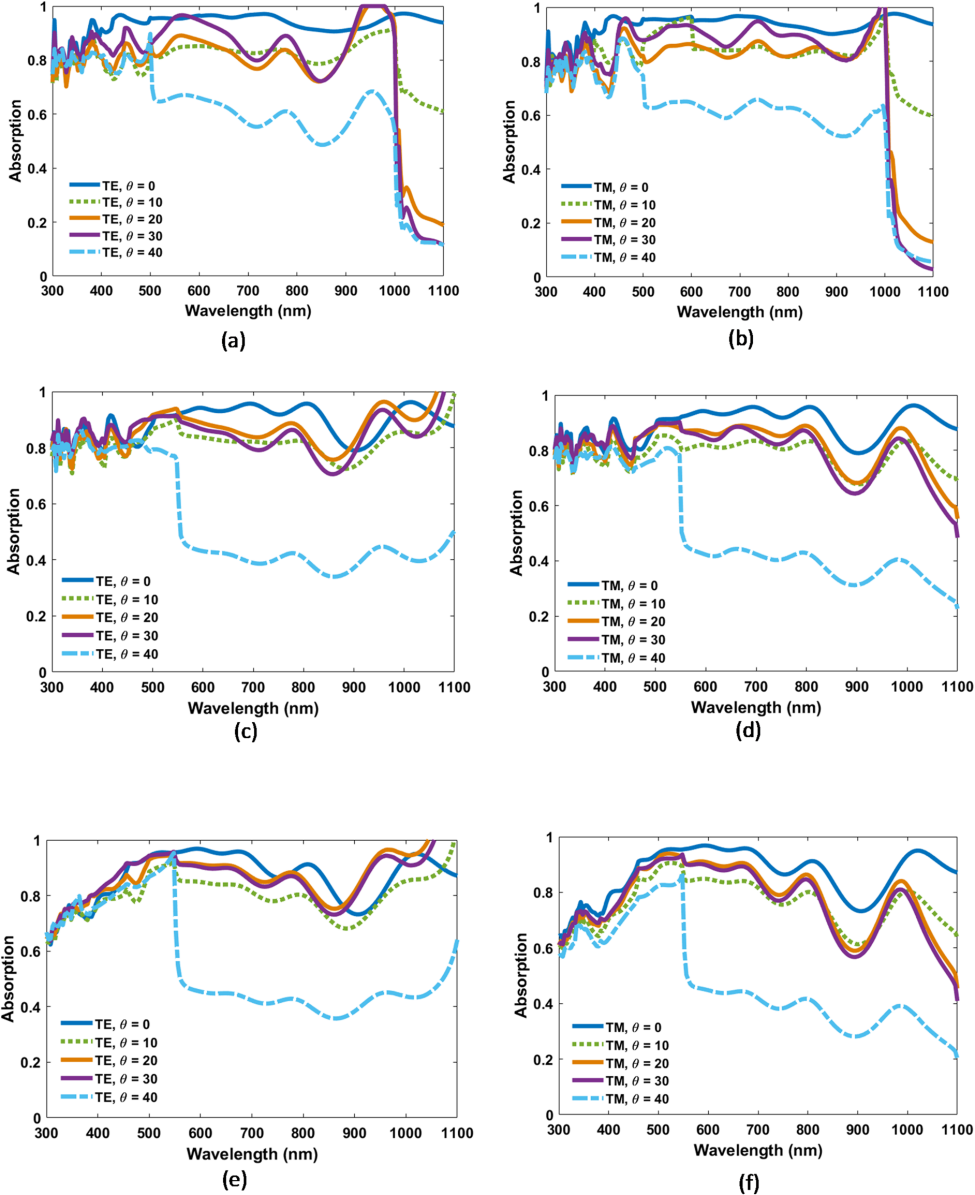
Absorption performance under varying incidence angles for the proposed CIGS TFSC including (**a**, **b**) Cubic nano-particles under TE and TM polarizations, respectively, (**c**, **d**) Cylindrical nano-particles under TE and TM polarizations, and (**e**, **f**) Spherical nano-particles under TE and TM polarizations.

As sunlight often strikes the solar cell at oblique angles, evaluating its optical response under such conditions is crucial for accurate performance assessment. This study examines how the angle of incidence influences light absorption. Light inclination can impact device performance by increasing reflectivity or reducing light coupling into the active layer at steeper angles. Figure 7 depict the absorption spectra of TFSC integrated with spherical, cylindrical, and cubic NPs, analyzed under TE and TM polarizations over the 300–1100 nm spectral range and across varying the angle of incidence. Table 2 summarizes the corresponding average optical absorption under TE and TM polarizations for incidence angles ranging from 0° to 40°. The results show that at normal incidence, all shapes exhibit high average absorption (> 88%), with the cubic design achieving the highest value (93.5% for both TE and TM) and demonstrating complete polarization insensitivity. As the incidence angle increases to 10°–20°, the cubic structure maintains balanced TE/TM (82.2% vs. 80% at 10°) absorption with only moderate losses, while the cylindrical and spherical shapes show greater polarization dependence, particularly with reduced TM absorption, as shown in Table 2. At 30°, cubic nanoparticles still preserve similar TE/TM values (~ 79%), whereas the other shapes exhibit larger TE–TM discrepancies. At 40°, the cubic design outperforms the others, retaining 60% and 59% absorption for TE and TM, respectively, compared to much lower values for cylindrical (53.2%/50.5%) and spherical (55%/48%) shapes. Notably, all NP configurations exhibit nearly identical performance within the 300–800 nm range under both TE and TM polarizations, indicating polarization insensitivity and the potential for enhanced TFSC efficiency. These results indicate that cubic nanoparticles provide the most robust broadband and angle-insensitive absorption, owing to their ability to support strong, multi-directional Mie resonances and maintain efficient light coupling into the absorber across a wide range of angles and polarizations. This superior angular stability makes the cubic configuration particularly advantageous for real-world solar energy harvesting, where sunlight is unpolarized and incident from varying directions throughout the day.

**Table 2:** Summary of average absorption for the proposed designs of TFSC under TE and TM polarizations, for various light incidence angles (0° to 40°).

Incidence angle (θ)	Average absorption (%)					
	Cubic		Cylindrical		Spherical	
	TE	TM	TE	TM	TE	TM
*0*	93.5	93.5	90.1	90.1	88.4	88.4
*10*	82.2	80	82	79	80.2	76
*20*	76.2	75.9	87.6	81.5	88.7	77.7
*30*	79	78.8	85.3	80	87.7	76.4
*40*	60	59	53.2	50.5	55	48

**Fig. 8:**
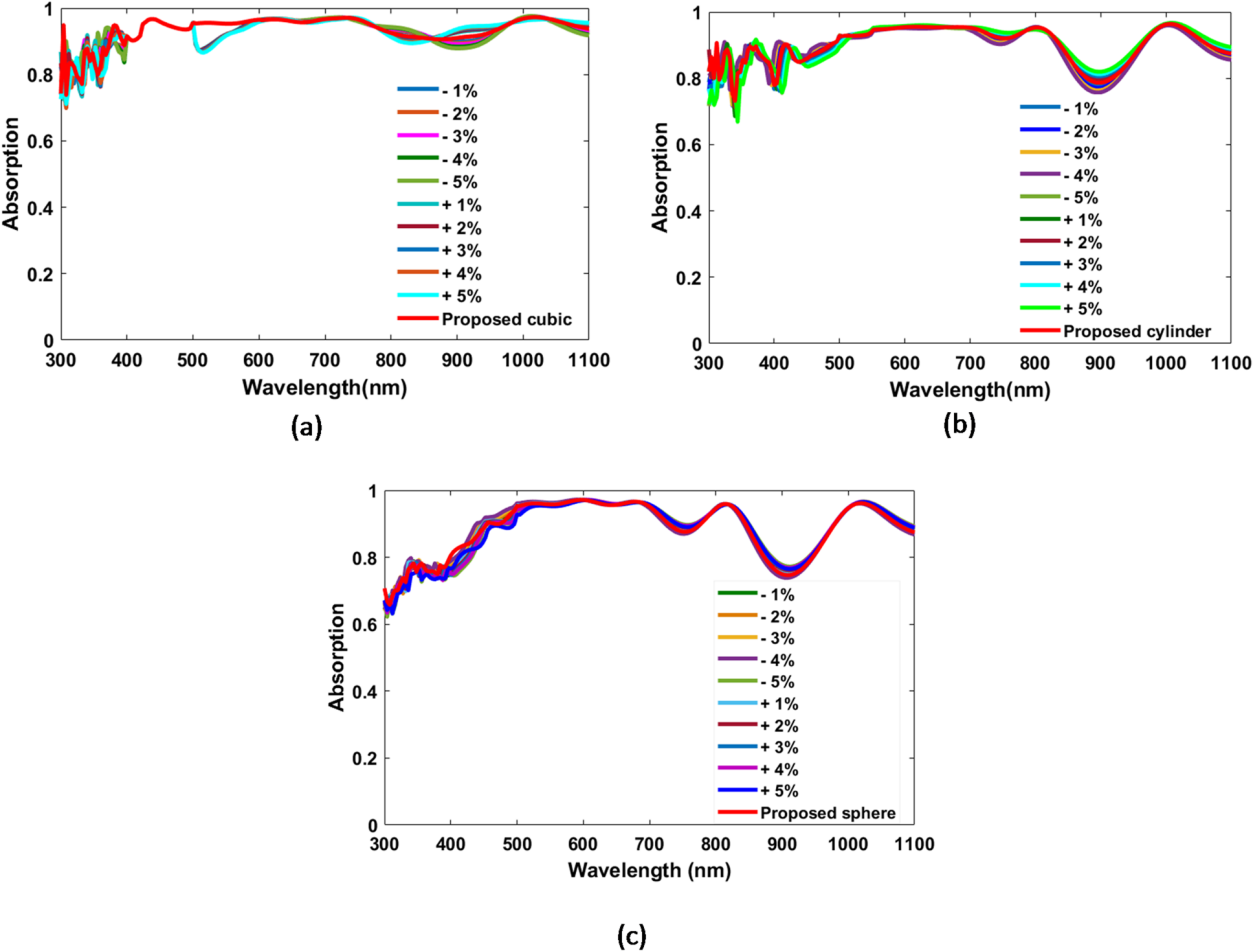
Absorption performance of the proposed CIGS-based TFSC including cubic, cylindrical, and spherical dielectric nano-particles with fabrication tolerances from ± 1% to ± 5% (**a**) Cubic, and (**b**) Cylindrical, and (**c**) Spherical.

Nanofabrication processes is inherently affected by size deviations due to technological limitations. Hence, a tolerance analysis is conducted for the optimized design incorporating cubic, cylindrical, and spherical AlAs dielectric nano-particles in order to assess the fabrication robustness of the suggested CIGS-based TFSC. Figure 8 illustrates the absorption spectra of the proposed TFSC with integrated NPs, considering simultaneous dimensional variations of all structural parameters within a tolerance range of ± 1% to ± 5%. Remarkably, the proposed structures sustain high absorption efficiency even when all optimized structural parameters are simultaneously varied by ± 5%.

The tolerance analysis is further extended to assess how small changes in each geometric dimension individually affect the device’s optical absorption. In this work, the tolerance of a specific parameter is studied while other dimensions of the design are held constant at their optimum values. Table 3 presents the average absorption of the proposed TFSC design with the suggested NPs, considering a ± 5% fabrication tolerance. It is noteworthy that the proposed TFSC configurations incorporating cubic, cylindrical, and spherical AlAs nanoparticles exhibit high average absorption levels of 93.3%, 90%, and 87.1%, respectively. This highlights the exceptional fabrication tolerance of the proposed TFSC design, which maintains strong optical performance despite minor dimensional deviations, confirming its robustness and practical manufacturability.

**Table 3:** Summary of fabrication tolerance for the proposed designs of TFSC.

Parameter	Tolerance	Average absorption (%)					
		Cubic		Cylindrical		Spherical	
		+ 5%	-5%	+ 5%	-5%	+ 5%	-5%
*L*_*1*_(nm)	*L*_*1*_ ± 5%	93.6	93.3	--	--	--	--
*L*_*2*_ (nm)	*L*_*2*_ ± 5%	93.5	93.5	--	--	--	--
*L*_*3*_ (nm)	*L*_*3*_ ± 5%	93.6	93.3	--	--	--	--
*d*_*1*_ (nm)	*d*_*1*_ ± 5%	93.55	93.6	90.1	90.1	87.2	87.2
*d*_*2*_ (nm)	*d*_*2*_ ± 5%	93.5	93.5	90.1	90	87.2	87.2
*h*_*1*_ (nm)	*h*_*1*_ ± 5%	93.5	93.5	90	90	--	--
*h*_*2*_ (nm)	*h*_*2*_ ± 5%	93.5	93.5	90	90	--	--
*h*_*3*_ (nm)	*h*_*3*_ ± 5%	93.5	93.5	90	90.1	--	--
*R*_*1*_ (nm)	*R*_*1*_ ± 5%	--	--	90.1	90.1	87.1	87.2
*R*_*2*_ (nm)	*R*_*2*_ ± 5%	--	--	90	90.1	87.1	87.1
*R*_*3*_ (nm)	*R*_*3*_ ± 5%	--	--	90.3	90	87.1	87.1
*H_ZnO*(nm)	*H_ZnO* ± 5%	93.6	93.5	90.3	90	87.2	87.2

**Fig. 9:**
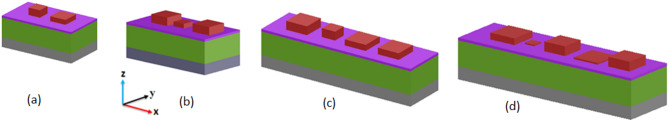
(**a**) 3D view of the proposed CIGS TFSC with the (**a**) two, (**b**) three, (**c**) four, and (**d**) five cubic dielectric nanoparticles.

**Fig. 10:**
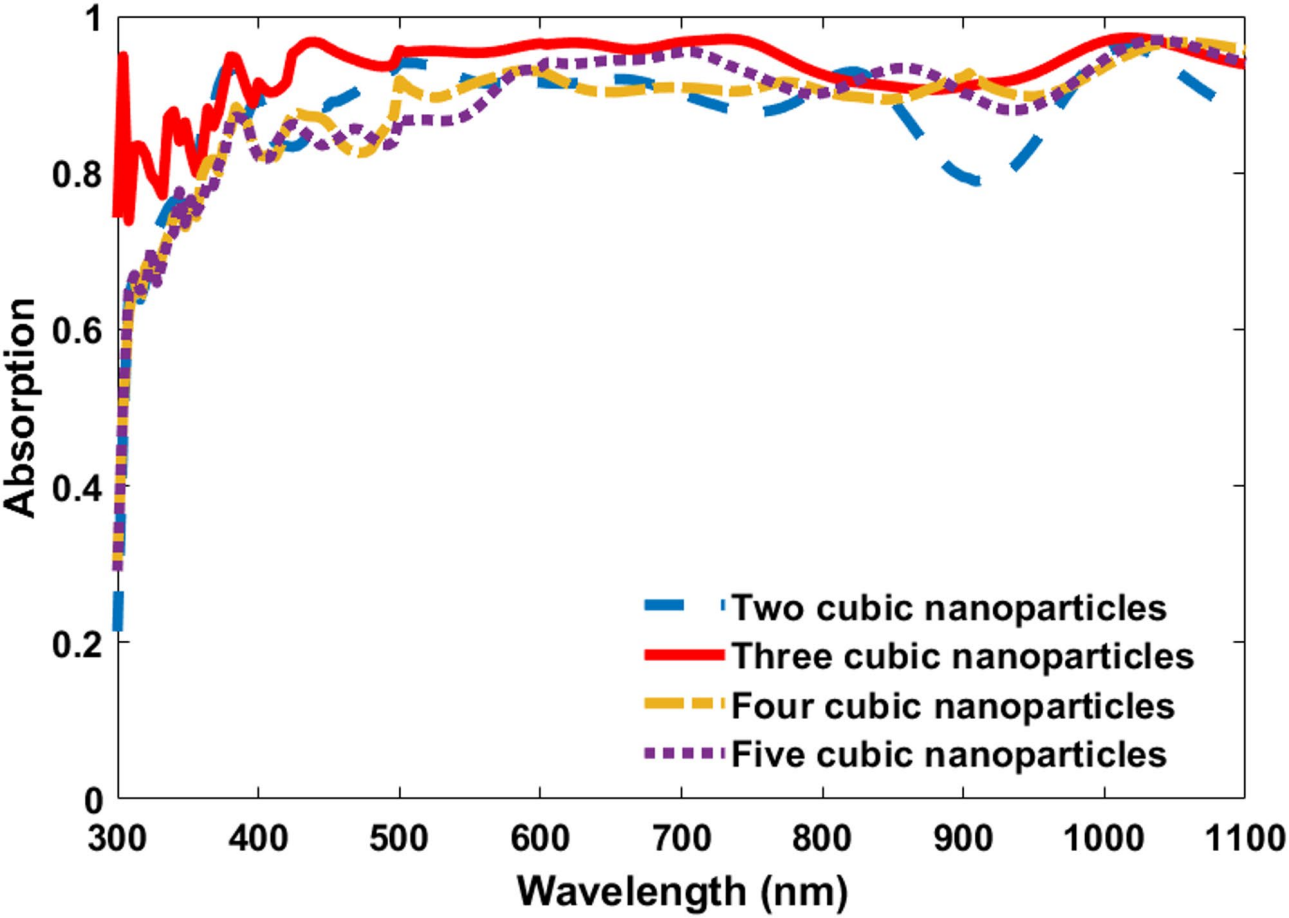
Comparison of the CIGS TFSC with two, three, four and five cubic dielectric nanoparticles atop it in the wavelength range 300–1100 nm.

Regarding the amount density of nanoparticle, additional optimization studies were conducted for the CIGS TFSC with cubic-shaped nanoparticles to assess the effect of the nanoparticle surface density on the optical absorption and J_ph_. Four cubic nanoparticle configurations were examined: two nanoparticles, three nanoparticles, four nanoparticles, and five nanoparticles arranged on the top surface of the CIGS solar cell, as shown in Fig. 9. The corresponding absorption in the wavelength range 300–1100 nm is presented in Fig. 10. The results demonstrated that the configuration with three cubic nanoparticles provided the most balanced and efficient optical response. Specifically, the photocurrent density reached 41.45 mA/cm² for three dielectric nanoparticles. When the number of nanoparticles was reduced to two, the photocurrent density decreased to 38.8 mA/cm², indicating insufficient light coupling at lower nanoparticle densities. Conversely, the Jsc is slightly decreased to 39.56 mA/cm² and 39.54 mA/cm², respectively, when the number of the nanoparticles was increased to four and five, respectively. This indicates that increasing nanoparticle density beyond the optimal point does not enhance device performance and may even introduce optical losses. Consequently, the three-nanoparticle configuration was identified as the most effective design, offering the highest absorption and photocurrent with minimal material usage. The corresponding values of the photocurrent density and periodicity are summarized in the following Table 4.

**Table 4:** Summary of photocurrent density for the CIGS TFSC with two, three, four and five cubic dielectric nanoparticles atop it in the wavelength range 300–1100 nm.

Configuration	Two cubic NPs	Three cubic NPs	Four cubic NPs	Five cubic NPs
Photocurrent density (mA/cm²)	*38.85*	*41.45*	*39.56*	*39.54*
*P*_*x*_ *× P*_*y*_	*800 nm ×500 nm*	*1000 nm ×500 nm*	*1600 nm ×500 nm*	*1800 nm ×500 nm*

### Electrical characterization

**Fig. 11:**
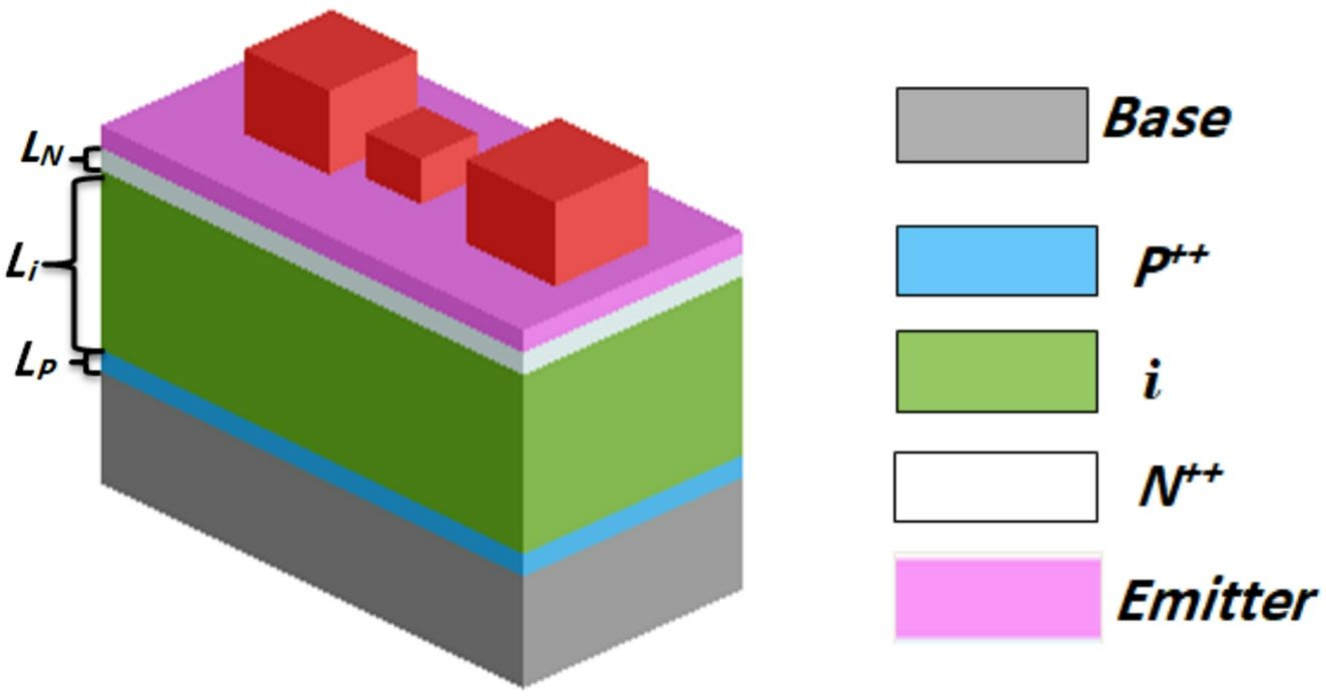
A schematic diagram of the reported TFSC incorporating AlAs dielectric nano-particles in cubic shape with p-i-n doping.

To evaluate the electrical performance of the reported TFSC structures, electrical simulations are performed for the three proposed CIGS-based solar cell with integrated NPs relative to the conventional CIGS-based solar cell with and without ARC. Figure 11 illustrates the architecture of the TFSC under investigation, consisting of a p-i-n (p-type/intrinsic/n-type) junction positioned on aluminum (Al) substrate. ZnO serves as the top electrode, while Al acts as the bottom contact. In this study, the P + + and N + + layer thicknesses (L_p_ and L_N_) are fixed at 40 nm during simulations. The doping concentrations for the p-type and n-type regions within the CIGS layer are set to 2 × 10^16^ cm⁻³ and 1 × 10^14^ cm⁻³, respectively^51^. Electron and hole mobilities are 100 cm^2^/V.s and 25 cm^2^/V.s, respectively. Carrier lifetimes in the P + and N + regions are defined as 50 ns, and 10 ns, respectively^52^. Further, the losses from the surface recombination (SR) and the internal radiative, Shockley-read-hall (SRH), and Auger are taken into consideration^24^. In this research, the surface recombination velocity (SRV) is assumed to be 1 × 10^7^ cm/s^51^. All electrical parameters of the CIGS are summarized in Table 5.

**Fig. 12:**
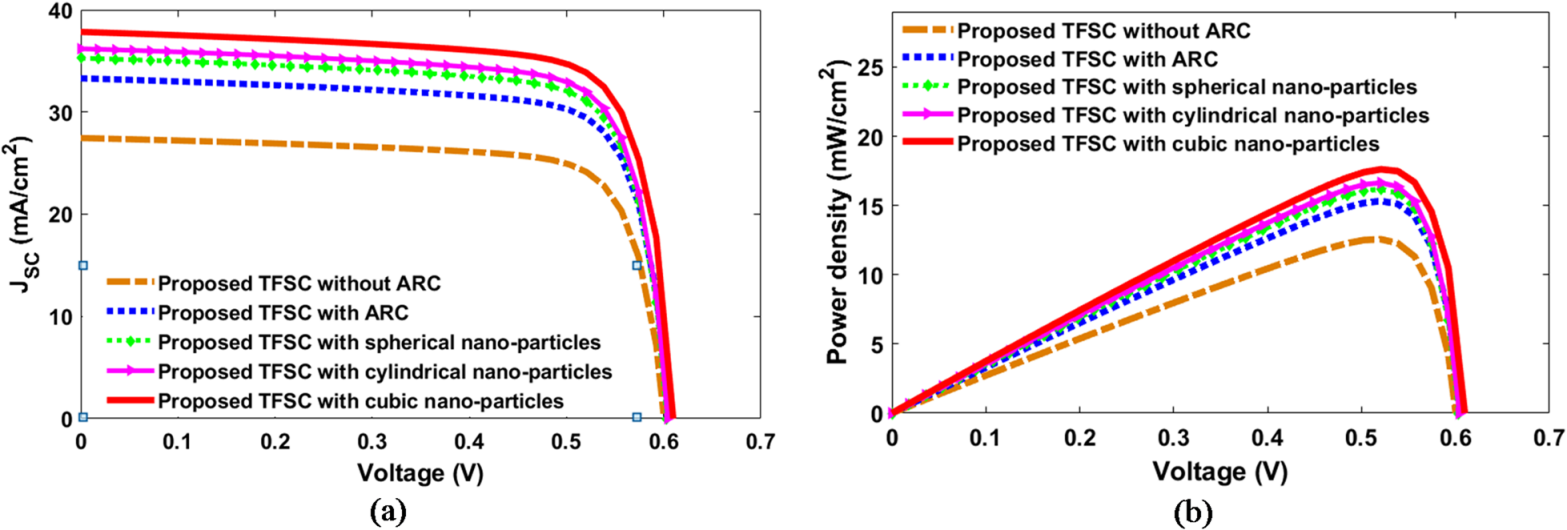
The short-circuit current density $\:{\mathrm{J}}_{\mathrm{S}\mathrm{C}}$ and power density of the proposed designs, (**a**) $\:{\mathrm{J}}_{\mathrm{S}\mathrm{C}}\:,$ and (**b**) Power density.

Figure 12(a) and (b) compare the electrical characteristics of the proposed designs containing different nanoparticle shapes with a conventional TFSC with and without ARC. The corresponding electrical outcomes including J_SC_, V_OC_, FF, and $\:{\upeta\:}$ are detailed in Table 6. The inclusion of an ARC increased J_SC_ and η to 33.32 mA/cm^2^ and 15.31% for both TE and TM polarizations, in comparison to 27.46 mA/cm^2^ and 12.56% of the traditional TFSC, respectively. Further enhancement is achieved by integrating dielectric NPs of diverse geometries, which promote enhanced light absorption. This leads to a higher number of photons being absorbed, resulting in increased generation of electron–hole pairs within the CIGS layer. Consequently, more photogenerated carriers are successfully collected at the electrodes, boosting the output current. Hence, J_SC_ rises directly with the increase in absorbed photon flux. In the spherical-based NPs, J_SC_ is increased to 35.27 mA/cm^2^ with a corresponding η of 16.18%. The design of cylindrical NPs further enhances performance, yielding η of 16.7% and J_SC_ of 36.2 mA/cm². Among all examined configurations, the cubic NP design exhibits superior electrical performance, delivering J_SC_ of 37.84 mA/cm² and η of 17.62% with an increase of 37.8% and 40.3%, respectively, when compared to the base TFSC. The.

**Table 5:** Electrical parameters of CIGS.

Parameters	CIGS
Thickness (nm)	400
Dielectric constant	13.6
Bandgap (eV)	1.04–1.67
Electron affinity (eV)	4.5
Electron mobility (cm^2^/Vs)	100^52^
Hole mobility (cm^2^/Vs)	25^52^
Conduction band effective density of states (cm^− 3^)	2.2 × 10^18^
Valence band effective density of states (cm^− 3^)	1 × 10^19^
Donor concentration (cm^− 3^)	1 × 10^14^ ^51^
Acceptor concentration (cm^− 3^)	2 × 10^16^ ^51^
Electron lifetime (s)	1 × 10^− 8^
Hole lifetime (s)	5 × 10^− 8^

enhanced electrical performance of the cubic design aligns with its increased broadband absorption and higher photocurrent density, as it facilitates more efficient light–matter interaction and deeper photon penetration into the active layer, leading to improved electron–hole pair generation throughout the entire absorber thickness. The observed gap between J_ph_ and J_SC_ results from various recombination mechanisms such as Shockley–Read–Hall, radiative, and Auger recombination. Furthermore, a slight rise in V_OC_ was noted.

**Table 6:** Comparison of electrical characteristics of the studied designs of TFSC.

Proposed Design	$\:{\mathbf{J}}_{\mathbf{s}\mathbf{c}}$ (mA/cm^2^)	$\:{\mathbf{V}}_{\mathbf{O}\mathbf{C}}\:\left(\mathbf{V}\right)$	η (%)	FF (%)
Baseline CIGS TFSC	27.46	0.60	12.56	76.2
CIGS TFSC with ARC	33.32	0.61	15.31	76.0
CIGS TFSC with spherical nano-particles	35.27	0.61	16.18	76.1
CIGS TFSC with cylindrical nano-particles	36.20	0.61	16.70	76.2
CIGS TFSC with cubic nano-particles	37.841	0.62	17.62	76.3

Finally, to evaluate the effectiveness of the proposed TFSC design, Table 7 provides a comparative overview of various nanostructured solar cell configurations reported in the literature. These designs incorporate different nano-photonic strategies to enhance light absorption and device efficiency. In this regard, Yang et al.^53^ proposed a 2 μm-thick thin-film C-Si SC featuring partially embedded dielectric spheres. The impact of embedding metal nanoparticle arrays has been studied with different geometries into the depletion region of TFSCs^54^. Additionally, Araujo’s team investigated the use of high-performance plasmonic back reflectors has been investigated to enhance light trapping in silicon TFSCs^55^. Further, a Se-based TFSC has been introduced incorporating Ti and Au metallic sublayers together with Au NPs^23^. Mohsin’s team employed a plasmonic nanoparticle array embedded within the silicon active layer of TFSCs, combined with an ARC and aluminum reflective layer^2^. Elrabiaey et al. proposed and investigated a TFSC design incorporating dielectric nanowires^24^. Shameli’s team studied two designs: one with a metallic fractal nano-carpet embedded in the silicon layer and another where the active layer itself follows a fractal pattern^6^. Chaudry’s team investigated light-trapping efficiency using semiconductor nanoparticle arrays on the top surface of thin-film GaAs solar cells^25^. Shaghouli et al. introduced an integrated structure combining silver fractal pattern on the top side of the active layer with leaky wave optical NAs on the bottom side of the absorber layer acting as back reflector^21^. Ahmed’s team introduced a TFSC combining front-side nanotexturing and rear-side amorphous silicon nanowires^28^. Deng’s team presented an advanced TFSC design featuring periodic plasmonic Ti NPs embedded in InP thin films^27^. Soudagar’s team investigated the effect of varying ZnO electron transport layer (ETL) thickness on the performance of CIGS TFSC^56^. Cao’s team revealed that the efficiency of carrier transport in Sb₂Se₃ TFSC is influenced by the absorber layer thickness^57^.

In contrast to prior works, the proposed 400 nm-thick CIGS-based architecture leverages simple, low-loss AlAs dielectric nanoparticles that induce strong localized optical fields. Among the tested geometries, the cubic-shaped nanoparticles deliver the most significant enhancement. The reported TFSC with cubic NPs achieves remarkable improvements in conversion efficiency exceeding designs in the literature by 44.4%, 19.86%, 43.3%, 10.8%, 29.4%, and 131.2 relative to Refs^2,23,24,28,54^, and^57^ respectively. Although Araujo’s design^55^ demonstrated a slightly higher efficiency, it required a much thicker 6600 nm absorber, while our design achieves high performance with only 400 nm—supporting reduced material use and mechanical flexibility. Similarly, compared to Deng’s design^27^, our TFSC shows 18.3% enhancement in J_SC_. It is worth noting that their approach presents two key limitations: first, embedding Ti nanoparticles within the absorber increases fabrication complexity; second, parasitic absorption from plasmonic NPs was not separated from the total absorption, potentially inflating the estimated active-layer efficiency. Furthermore, J_SC_ observed in this research shows substantial gains of 32.3%, 65.9%, 30.1%, 57.67%, 57.8%, 22.4%, 147.9%, 95.7%, 286.1%, 63%, 18.3%, 7.8%, and 23.2%, respectively.

**Table 7:** Comparison of electrical performance metrics of the TFSCs in this study with those reported in literature.

Ref.	$\:{\mathbf{J}}_{\mathbf{s}\mathbf{c}}$ (mA/cm^2^ )	$\:{\mathbf{V}}_{\mathbf{O}\mathbf{C}}\:\left(\mathbf{V}\right)$	η (%)	FF (%)
2016^53^	28.6	--	--	--
2016^54^	22.8	0.65	12.2	83
2018^55^	29.09	0.95	23.01	83.47
2018^23^	24	1	14.7	67
2020^2^	23.98	0.61	12.3	84
2020^24^	30.91	0.623	15.9	83.01
2021^6^	15.26	--	--	--
2022^25^	19.34	--	--	--
2023^21^	9.8	--	--	--
2024^28^	23.22	0.749	13.62	78.29
2024^27^	31.996	1.053	29.634	87.89
2025^56^	35.1	0.81	--	--
2025^57^	30.71	--	7.62	--
Proposed TFSC with spherical nano-particles	35.27	0.61	16.18	76.1
Proposed TFSC with cylindrical nano-particles	36.20	0.61	16.70	76.2
Proposed TFSC with cubic nano-particles	37.841	0.62	17.62	76.3

**Fig. 13:**
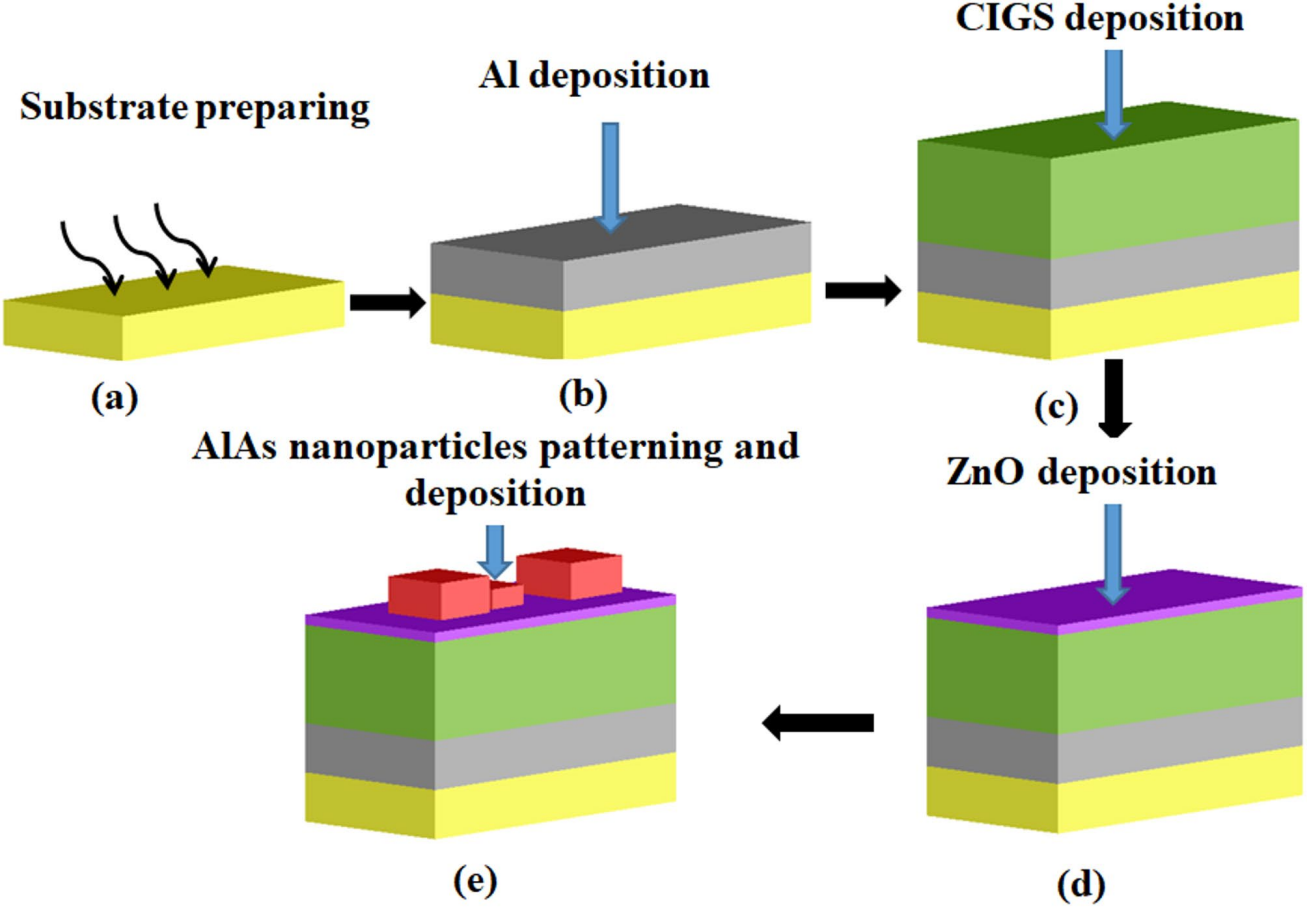
Schematic illustration of the fabrication process for the proposed CIGS-based TFSC with AlAs cubic nanoparticles on the top layer. The process involves: (1) substrate preparation through ultrasonic cleaning and oxygen plasma treatment, (2) deposition of a 200 nm Al back reflector via RF magnetron sputtering, (3) RF magnetron sputtering of a quaternary CIGS absorber layer to form a high-quality chalcopyrite film, (4) deposition of ZnO front electrode by RF sputtering, (5) cubic nanoparticles patterning using GS-EBL and deposition using RF magnetron sputtering.

The fabrication process of the proposed CIGS-based TFSC incorporating cubic AlAs NPs atop ARC, can be achieved as shown in Fig. 13. Initially, the substrate should be cleaned using ultrasonic baths in acetone, isopropanol (IPA), and deionized water. This is followed by an oxygen plasma treatment to remove residual organic contaminants and enhance adhesion for subsequent layers^56^. A 200 nm Al back reflector is then deposited via RF magnetron sputtering under 100 W RF power, 0.5 Pa argon gas with a deposition rate of 300 nm/min^56^. Next, the CIGS absorber layer (~ 400 nm thick) will be then deposited using RF magnetron sputtering at 400 °C^56,58^. To achieve a uniform composition and a high-quality chalcopyrite phase, a quaternary CIGS target can be utilized. In the RF magnetron sputtering process, a radiofrequency plasma is sustained in an inert argon atmosphere, causing atoms to be ejected from the target material and subsequently deposited as a thin film on the substrate^56^. This deposition technique offers excellent control over film thickness, stoichiometry, and uniformity, making it highly suitable for producing complex multilayer TFSC architectures^56,59,60^. After that, a 40 nm of ZnO will be deposited atop the CIGS absorber via RF sputtering at temperatures below 150 °C to ensure compatibility with underlying layers^61^. For the cubic dielectric NPs, a combination of lithographic patterning, precise film deposition, and reactive-ion etching (RIE) can be utilized^62^. In this respect, an AlAs dielectric layer is deposited via RF magnetron sputtering with a thickness slightly exceeding the tallest nanoparticle to ensure uniform coverage and surface integrity. The NP pattern is defined using grayscale electron-beam lithography (GS-EBL), which modulates the electron-beam dose to produce varying resist thicknesses in a single exposure^63,64^. It is worth noting that a negative-tone resist (AZ nLOF 2070) was spin-coated at 3000 rpm for 40 s, forming a 170 nm AlAs layer, and soft-baked at 110 °C for 60 s^64^. Doses ranging from 12 to 32 µC/cm² were segmented into three calibrated levels to correspond with the suggested cube heights. Post-exposure, the sample was baked at 110 °C for 90 s and developed in 0.26 N TMAH for 60 s, producing a 3D resist profile with three different height levels^64^. Finally, this resist structure should be etched into the dielectric layer using RIE with a CHF₃/O₂ gas mixture (40 sccm CHF₃ and 2 sccm O₂), an RF power of 150 W, and a chamber pressure of 30 mTorr^65^. With an etch rate of approximately 2.8 nm/s, the grayscale profile will be accurately transferred into the dielectric, yielding final cube heights which are controlled through etching durations. Following the deposition of all layers, the devices underwent post-deposition annealing at 550 °C, in a controlled environment. This step aims at improving crystallinity, strengthening interfacial bonding, and enhancing the overall performance of the CIGS-based TFSC^56^. Final device contacts will be established via solder pads on the Al rear contact and wire bonding to the front grid. The entire structure may be encapsulated using a transparent epoxy and glass or polymer superstrate to protect the device.

## Conclusion

This study presents an optimized CIGS TFSC design incorporating AlAs dielectric nanoparticles; cubic, cylindrical, and spherical, on the top surface to reduce reflection and enhance light absorption. Using a 3D FDTD approach and optimizing geometrical parameters via PSO, the cubic nanoparticles demonstrates superior broadband absorption (> 97%), achieving 31.7% improvement over the baseline. The electrical simulations reveal the highest J_SC_ (37.84 mA/cm²) and efficiency (17.6%), representing enhancements of 37.8% and 40.3%, respectively. The proposed design effectively preserves the internal planar CIGS structure, minimizing recombination and fabrication complexity of nanostructures-based active material. However, the work remains simulation-based and does not account for thermal stability under real operating conditions. Future work will involve experimental validation of the proposed design by fabricating and characterizing CIGS-based solar cells with integrated AlAs nanoparticles. The optimization framework will also be extended to include hybrid dielectric–plasmonic structures to further improve light trapping and device performance.

## Data Availability

All data in support of the findings of this paper are available within the article.
